# Elevations in blood glucose before and after the appearance of islet autoantibodies in children

**DOI:** 10.1172/JCI162123

**Published:** 2022-10-17

**Authors:** Katharina Warncke, Andreas Weiss, Peter Achenbach, Thekla von dem Berge, Reinhard Berner, Kristina Casteels, Lidia Groele, Konstantinos Hatzikotoulas, Angela Hommel, Olga Kordonouri, Helena Elding Larsson, Markus Lundgren, Benjamin A. Marcus, Matthew D. Snape, Agnieszka Szypowska, John A. Todd, Ezio Bonifacio, Anette-G. Ziegler, for the GPPAD and POInT Study Groups

**Affiliations:** 1 Institute of Diabetes Research, Helmholtz Munich, German Center for Environmental Health, Munich, Germany.; 2 Department of Pediatrics, Kinderklinik München Schwabing, School of Medicine, Technical University Munich, Munich, Germany.; 3 Forschergruppe Diabetes, School of Medicine, Klinikum Rechts der Isar, Technical University Munich, Munich, Germany.; 4 Forschergruppe Diabetes e.V. at Helmholtz Munich, German Research Center for Environmental Health, Munich, Germany.; 5 Kinder- und Jugendkrankenhaus auf der Bult, Hannover, Germany.; 6 Department of Pediatrics, University Hospital Carl Gustav Carus, Technische Universität Dresden, Dresden, Germany.; 7 Department of Pediatrics, University Hospitals Leuven, Leuven, Belgium.; 8 Department of Development and Regeneration, KU Leuven, Leuven, Belgium.; 9 Department of Paediatrics, The Children’s Clinical Hospital Józef Polikarp Brudziński, Warsaw, Poland.; 10 Institute of Translational Genomics, Helmholtz Munich, German Research Center for Environmental Health, Neuherberg, Germany.; 11 Technische Universität Dresden, Center for Regenerative Therapies Dresden, Dresden, Germany.; 12 Paul Langerhans Institute Dresden of the Helmholtz Munich at University Hospital Carl Gustav Carus and Faculty of Medicine, Technische Universität Dresden, Germany.; 13 Unit for Pediatric Endocrinology, Department of Clinical Sciences Malmö, Lund University, Malmö, Sweden.; 14 Department of Paediatrics, Skåne University Hospital, Malmö, Sweden.; 15 Department of Pediatrics, Kristianstad Hospital, Kristianstad, Sweden.; 16 Oxford Vaccine Group, University of Oxford Department of Paediatrics, and NIHR Oxford Biomedical Research Centre, University of Oxford, Oxford, United Kingdom.; 17 Department of Paediatrics, Medical University of Warsaw, Warsaw, Poland.; 18 Wellcome Centre for Human Genetics, Nuffield Department of Medicine, NIHR Oxford Biomedical Research Centre, University of Oxford, Oxford, United Kingdom.; 19 The GPPAD and POInt study groups are detailed in the Supplemental Acknowledgments.

**Keywords:** Immunology, Diabetes, Insulin

## Abstract

The etiology of type 1 diabetes has polygenic and environmental determinants that lead to autoimmune responses against pancreatic β cells and promote β cell death. The autoimmunity is considered silent without metabolic consequences until late preclinical stages,and it remains unknown how early in the disease process the pancreatic β cell is compromised. To address this, we investigated preprandial nonfasting and postprandial blood glucose concentrations and islet autoantibody development in 1,050 children with high genetic risk of type 1 diabetes. Pre- and postprandial blood glucose decreased between 4 and 18 months of age and gradually increased until the final measurements at 3.6 years of age. Determinants of blood glucose trajectories in the first year of life included sex, body mass index, glucose-related genetic risk scores, and the type 1 diabetes–susceptible *INS* gene. Children who developed islet autoantibodies had early elevations in blood glucose concentrations. A sharp and sustained rise in postprandial blood glucose was observed at around 2 months prior to autoantibody seroconversion, with further increases in postprandial and, subsequently, preprandial values after seroconversion. These findings show heterogeneity in blood glucose control in infancy and early childhood and suggest that islet autoimmunity is concurrent or subsequent to insults on the pancreatic islets.

## Graphical abstract

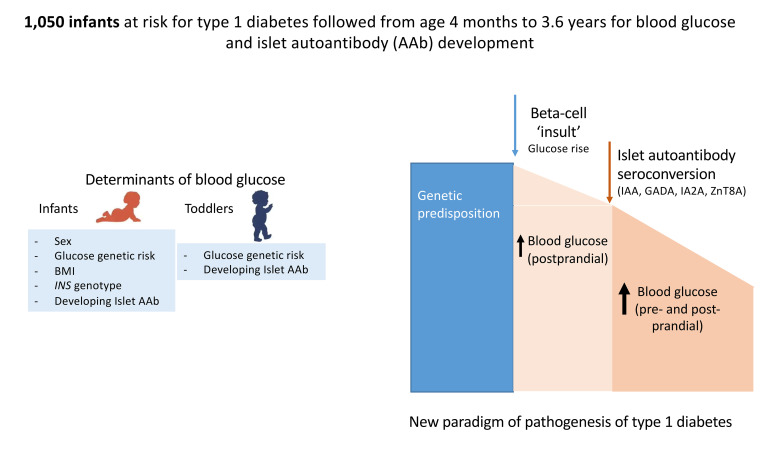

## Introduction

The supply, uptake, and metabolism of glucose are cornerstones of energy production for cell and organ function and survival. Blood glucose levels are regulated by a multitude of hormones and factors, but ultimately by the response of pancreatic islet cells to the glucose concentration and the abilities of cells and tissues to uptake and use glucose (1). In adults, genome-wide association studies have identified numerous SNPs that determine fasting blood glucose levels and homeostatic model assessment of β cell function (HOMA-B), indicating genetic variation in glucose homeostasis (2, 3).

Disrupted glucose homeostasis leads to diabetes, of which there are multiple forms and etiologies (4). Type 1 diabetes (T1D) is the most frequent form in children and is characterized by the appearance of autoimmunity against the pancreatic islets. The first years of life are a crucial period for the initiation of this autoimmunity, with a peak incidence occurring at 12 months of age (5, 6). This is also a crucial developmental period for the pancreas and pancreatic islets as well as organs, such as the brain, that are strongly dependent upon glucose homeostasis for their function (1, 7). Despite the potential relevance of early childhood metabolism on health, relatively little is known about longitudinal glucose homeostasis in the first years of life and its potential relationship to T1D development.

The traditional model of T1D pathogenesis proposes that genetically susceptible individuals develop chronic islet autoimmunity, which eventually causes abnormal glucose tolerance and the need for insulin replacement, a process that can take years or decades (8, 9). This dogma is based on longitudinal follow-up of islet autoantibody–positive individuals. However, little or no data on glucose control are available in children around the initiation of islet autoimmunity, and it remains unknown when the β cell first becomes affected or whether the β cell is involved in the etiological causes of the disease. Here, we examined the glucose concentrations in over 1,000 participants in the Primary Oral Insulin Trial (POInT) (10), from whom random glucose values were measured in venous blood samples that were collected in a prospective manner, starting from 4 to 7 months of age. We determined the blood glucose trajectories from this early age and examined the influence of genetics, BMI, demographic factors, and islet autoimmunity on glucose levels and age-related glucose trajectories. The findings indicate that metabolic shifts are present much earlier in the disease process than previously considered and that they may precede or be concurrent to the appearance of autoimmunity.

## Results

### Blood glucose concentrations and age trajectories during early childhood.

A total of 5,952 preprandial blood glucose concentrations were measured in 1,050 children (533 males) participating in POInT between 4 months and 3.6 years of age (Table 1 and Supplemental Table 1; supplemental material available online with this article; https://doi.org/10.1172/JCI162123DS1). Preprandial glucose concentrations were measured up to 9 times in each child (median, 6 times; IQR, 4–7 times), assessed at each study visit. Children had a median age of 0.51 years (IQR, 0.45–0.54 years) at the first measurement and followed up with preprandial blood glucose measurements for a median of 1.5 years (IQR, 0.9–2.0 years; Table 1 and Supplemental Table 1). From baseline to 1.35 years of age, postprandial blood glucose measurements (*n =* 3,068 in 1,045 children) were also available at 30 and 60 minutes after intake of the POInT investigational medicinal product (IMP), which was administered with a small quantity of food. The glucose genetic risk score (GRS) was calculated in 751 children with available genotyping (Supplemental Table 2). The median score was 16.8 (IQR, 14.9–18.8; Supplemental Figure 1), which is similar to the scores reported in adults (2).

**Figure 1:**
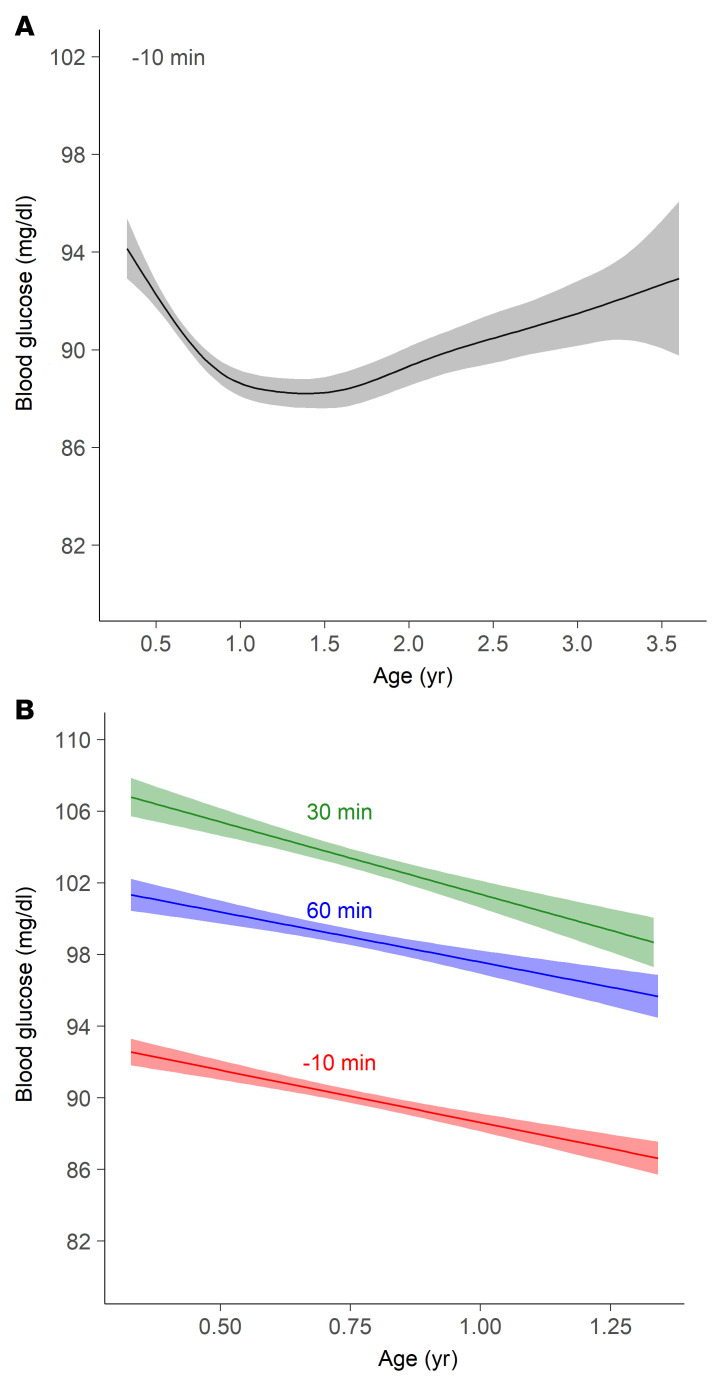
Pre- and postprandial blood glucose concentrations in relation to age. (**A**) Preprandial nonfasting glucose concentrations (10 minutes before food intake) modeled using a general additive model with thin-plate splines. The model was developed using 5,952 measurements in 1,050 children. (**B**) Glucose concentrations in relation to age by linear regression before food intake (−10 minutes; red; *n* = 3,966 measurements from 1,050 children) and at 30 minutes (green; *n* = 3,608 measurements from 1,045 children) and 60 minutes (blue; *n* = 3,267 measurements from 1,042 children) after food intake.

Modeling of site-normalized preprandial blood glucose concentrations produced a U-shaped curve with a nadir between 1 and 1.5 years of age (Figure 1A). The glucose concentrations were highest at the baseline measurement and decreased from a mean of 94.1 mg/dL (5.2 mmol/L) at 4 months of age to a mean of 88.2 mg/dL (4.9 mmol/L) at nadir, and increased thereafter to a mean of 92.6 mg/dL (5.1 mmol/L) at 3.5 years of age (Supplemental Table 1). The postprandial blood glucose values at 30 and 60 minutes were higher than the preprandial value (*P* < 0.0001; Supplemental Table 1). Linear regression showed a similar decline with age until 1.35 years for the preprandial and postprandial measurements (Figure 1B).

### Factors associated with blood glucose values.

The unexpected decline in pre- and postprandial blood glucose observed until 1 to 1.5 years of age and the subsequent rise at later ages prompted us to examine the potential determinants of the blood glucose values separately for infants (visits 1 to 4; age range 4 months to 1.35 years) and toddlers (visits 5 to 9; age range 1.39 to 3.5 years; Table 2 and Table 3). Factors influencing blood glucose in infants included sex (*P* < 0.0001), BMI (*P* < 0.0001), glucose GRS (*P* = 0.0031), and the genotype of the T1D susceptibility gene insulin (*INS*) (*P* = 0.00048) for preprandial values and sex (*P* < 0.0001) and glucose GRS (*P =* 0.0088) for postprandial values. The blood glucose in toddlers was associated with the glucose GRS (*P =* 0.0011). All factors that were associated with blood glucose remained significant in the multivariable analysis (Supplemental Tables 3 and 4). The associations also remained in a sensitivity analysis after excluding children from Sweden and the United Kingdom, where blood glucose values were measured using the HemoCue method (Supplemental Table 5). Sex, BMI, and the *INS* genotype were significant determinants of preprandial blood glucose values when only concentrations at the first study visit were considered (Supplemental Table 6).

**Figure 2:**
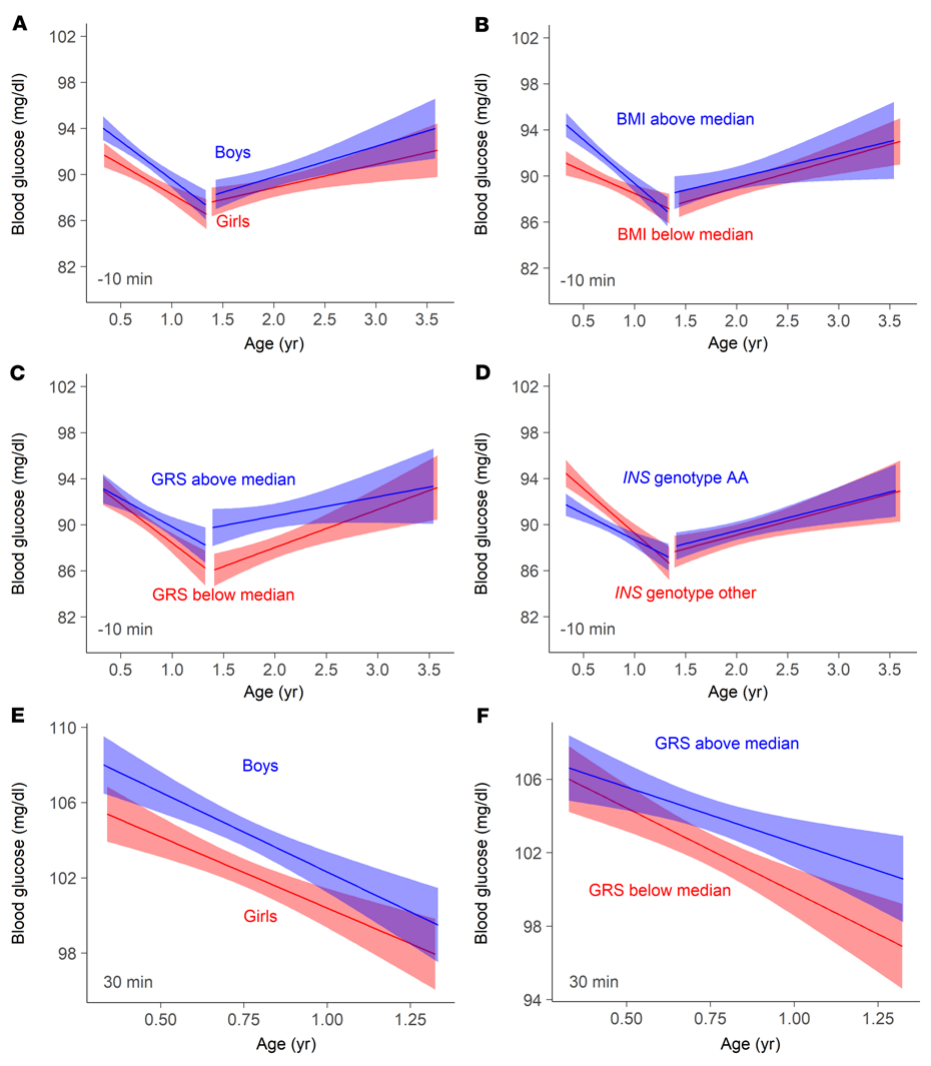
Factors associated with blood glucose concentration in early childhood. Linear regression of preprandial (10 minutes before food intake, **A**–**D**) and postprandial glucose values (30 minutes after intake, **E**–**F**) by visit age in the infancy period (visits 1–4; 4 months–1.35 years of age) and the toddler period (visits 5–9; 1.5–3.6 years of age). Analyses were separated by sex (**A** and **E**; boys [blue] vs. girls [red]), BMI (**B**; above the median [blue] vs. below the median [red]), glucose GRS (**C** and **F**; above the median [blue] vs. below the median [red]), and by *INS* genotype (**D**; AA [blue] vs. other [red]). Comparisons of regression coefficients between groups were performed using Student’s *t* test.

At the earliest measurements (4 months of age), the preprandial and postprandial blood glucose concentrations were 2.4 mg/dL (0.1 mmol/L) and 2.6 mg/dL (0.1 mmol/l) higher, respectively, in boys than in girls (Figure 2, A and E) and preprandial blood glucose concentrations were 3.3 mg/dL (0.2 mmol/L) higher in children with a BMI above the median than in children with a BMI below the median (Figure 2B). Convergence of blood glucose values was observed with age between boys and girls and between the high and low BMI groups. Blood glucose concentrations in children born to mothers with T1D were similar to concentrations in other children across all ages (Supplemental Figure 2).

Pre- and postprandial concentrations diverged between the high- and low-glucose GRS groups with increasing age (Figure 2, C and F, and Supplemental Table 6). Preprandial concentrations were around 4 mg/dL (0.2 mmol/L) higher in the high GRS group at age 1.5 years. The glucose GRS was not associated with BMI in early childhood (*r* = −0.01, *P* = 0.24). In addition to the genes previously reported to be associated with blood glucose concentrations, we examined the *INS* genotype because this is a risk factor for T1D (11) and is associated with variations in pancreatic insulin mRNA expression (12, 13). At the earliest measurements, the blood glucose values were 3 mg/dL (0.2 mmol/L) lower in children with the T1D-susceptible *INS* genotype AA than in children with nonsusceptible genotypes (Figure 2D). Values in the 2 groups converged, and no differences were observed after 1 year of age.

### Glucose values and islet autoimmunity.

The appearance of islet autoantibodies is associated with a marked increment in T1D risk and signals a detectable start of disease pathogenesis, after which abnormal glucose tolerance and clinical disease usually take several years to develop (14). Surprisingly, very early increases in blood glucose values were observed in children who developed islet autoantibodies during follow-up (Table 2 and Table 3). In particular, postprandial glucose in the infancy period differed between children who developed any (*P* = 0.00027) or multiple (*P* = 0.00059) islet autoantibodies during follow-up and children who remained islet autoantibody negative, while preprandial blood glucose during the toddler period was increased in children who developed any (*P* = 0.00077) or multiple (*P* = 0.00042) islet autoantibodies.

**Figure 3:**
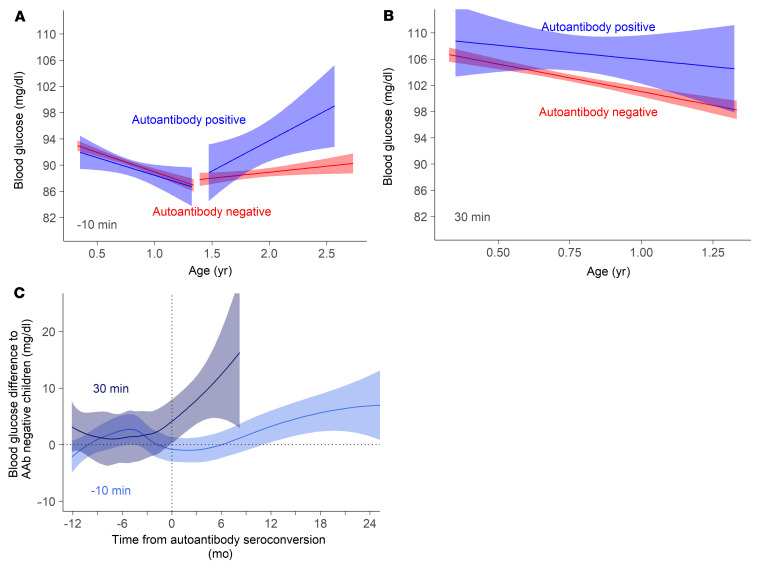
Pre- and postprandial blood glucose concentrations in relation to age and islet autoantibody development. (**A**) Linear regression of preprandial glucose values (10 minutes before food intake) by visit age in the infancy period (visits 1–4; 4 months–1.35 years of age) and the toddler period (visits 5–9; 1.5–3.6 years of age) in islet autoantibody–positive children (blue; *n* = 443 measurements from 77 children) and autoantibody-negative children (red; *n* = 5,242 measurements from 973 children). (**B**) Linear regression of postprandial blood glucose values at 30 minutes after food intake during the infancy period in islet autoantibody–positive children (blue) and autoantibody-negative children (red). (**C**) Locally weighted scatterplot smoothing (LOESS) of preprandial blood glucose values (10 minutes before food intake; light blue) and postprandial blood glucose values (30 minutes after food intake; dark blue) in islet autoantibody–positive children in relation to the timing of islet autoantibody appearance (seroconversion), where blood glucose is expressed as the difference from the mean value corrected by age in islet autoantibody–negative children. The horizontal dotted line indicates no difference (0), and the vertical dotted line corresponds to the timing of seroconversion.

The linear regression curves showed that children who developed islet autoantibodies experienced a steep rise in blood glucose values after the nadir and that the preprandial blood glucose values were significantly higher in islet autoantibody–positive children from around 1.8 years of age (Figure 3A). The preprandial blood glucose concentrations at 1.8 years were 4 mg/dL (0.2 mmol/L) higher in children who developed islet autoantibodies than in children who were negative. Moreover, the 30-minute postprandial concentrations were around 6 mg/dL (0.3 mmol/L) higher already at 9 months of age in children who developed islet autoantibodies (*P* < 0.0001; Figure 3B), indicating that there may be a difference in the ability to sense or dispose of blood glucose very early in life in children who develop islet autoimmunity and T1D. Differences were no longer significant at the 60-minute postprandial measurement (Supplemental Figure 3).

The majority (55/77) of islet autoantibody–positive children developed autoantibodies prior to 1.8 years of age (median age of first detection, 1.2 years; IQR, 0.8–1.5). Therefore, we examined the blood glucose values in relation to the time point of detection of islet autoantibodies (seroconversion) (Figure 3C). To obtain a measure of glucose values in the autoantibody-positive children relative to the autoantibody-negative children, the preprandial and 30-minute postprandial glucose values in the positive children were expressed as the difference relative to autoantibody-negative children at the age of measurement as described in Supplemental Figure 4. The values in the children who developed islet autoantibodies were not different from those in islet autoantibody–negative children 1 year prior to seroconversion (Figure 3C). However, postprandial blood glucose values started to diverge in the children who developed islet autoantibodies at around 2 months prior to islet autoantibody seroconversion. Values continued to rise after seroconversion and were 15.8 mg/dL above those in the islet autoantibody–negative children at 8 months after seroconversion. Divergence in preprandial blood glucose values was observed in the children who developed islet autoantibodies at around 9 months after seroconversion. This suggests that the development of islet autoimmunity is concurrent and perhaps preceded by a relative disturbance in the ability to control glucose.

## Discussion

Measurement of pre- and postprandial blood glucose concentrations in children at increased risk of developing T1D revealed a dynamic process of glucose regulation during infancy and early childhood. The blood glucose values were highest in infancy and decreased to a nadir at 12 to 18 months of age; trajectories were influenced by sex, BMI, and genetic factors, including the T1D susceptibility gene *INS*. Increased blood glucose concentrations were observed in children who developed islet autoantibodies. A rise in the 30-minute postprandial blood glucose occurred shortly prior to autoantibody seroconversion, with further increases in postprandial and subsequent rises in preprandial values shortly after seroconversion. These findings suggest that the onset of early islet autoimmunity is associated with insults or changes to the pancreatic islets that disturb glucose regulation.

This is the first study, to our knowledge, to longitudinally monitor and investigate blood glucose levels in a large number of infants and toddlers at increased genetic risk for T1D. The frequent measurements of blood glucose concentrations in over 1,000 children from as early as 4 months of age allowed us to identify small but significant differences in nonfasting pre- and postprandial blood glucose values within the normoglycemic range. The robustness of the findings is supported by our ability to identify heterogeneity associated with the glucose GRS, which was previously established from fasting blood glucose in adults; the results of the sensitivity analyses in which we excluded measurements from countries using alternative methods to measure blood glucose; and the relative consistency of the associations observed for both pre- and postprandial blood glucose values.

An unexpected finding was the decline in pre- and postprandial blood glucose values through to 12 to 18 months of age. It is widely considered that blood glucose values stabilize within a few days of life (15). However, our findings indicate that the glucose sensing and/or uptake machinery undergoes changes throughout infancy and early childhood, with the steepest changes occurring in the first year of life. Moreover, factors such as sex, BMI, and the *INS* genotype were associated with differences in blood glucose values in infancy, but not beyond 1 year of age. In rodents, profound changes in pancreatic islets and the pancreas occur in the first weeks and months after birth (16–18). The limited studies in humans indicate that some of these changes, including a reduction in islet β cell turnover and increased β cell maturation and variable expression of glucokinase, glucose transporters, and K^+^_ATP_ channels on fetal and neonatal β cells occur during the first year of life (19, 20). Furthermore, reduced blood glucose levels in this period were observed in girls and in children with the *INS* AA genotype, which is consistent with previous studies showing increased insulin responses in females (21) and increased *INS* mRNA expression in the pancreas from donors with the *INS* AA genotype (13). Therefore, a greater number of islets with better functional maturity that are able to produce a greater amount of insulin may represent an improvement in the ability to control blood glucose and contribute to the observed decline in blood glucose. It is also possible that, over the first year of life, there is a decline in the extrinsic glucose load and/or intrinsic glucose production due to the changes in diet and the needs of developing organs, such as the brain, which is dependent on the supply of glucose. Indeed, early studies that measured glucose production rates in children showed that brain size was the most important regulatory factor governing hepatic glucose production (22, 23). The blood glucose concentration is also determined by glucose uptake, which is influenced by insulin sensitivity. Although some studies have linked birth size to insulin sensitivity in childhood (24), relatively little is known about changes in insulin sensitivity during the first year of life. Greater BMI is associated with decreased insulin sensitivity (25) and higher glycated hemoglobin concentrations (26), which is consistent with our finding that higher BMI was associated with increased preprandial blood glucose until 1 year of age. However, BMI was not associated with postprandial blood glucose or with blood glucose values in toddlers.

A remarkable finding of our study is that elevated blood glucose values were observed close to islet autoantibody seroconversion. Although it is known that blood glucose and HbA1c rise prior to the onset of clinical T1D (27, 28), it is not yet understood when changes may occur in relation to the appearance of islet autoimmunity. Therefore, it was surprising to observe modest increases in the postprandial blood glucose values already shortly prior to seroconversion and further increases in both post- and preprandial values after seroconversion. These increases were unlikely to result from occasional measurements in samples when children had dysglycemia or diabetes, as such samples were excluded in these analyses. The increases are consistent with a direct contribution to the early peak incidence of islet autoimmunity around 1 year of age by events in the pancreas and/or disturbed glucose metabolism. The increased and rising pre- and postprandial values observed after seroconversion suggest that there is a sustained impairment in glucose disposition that exists very early in the autoimmune process and may be due to reduced islet function. This finding is consistent with the reduced pancreas volumes observed in islet autoantibody–positive individuals (29–31). Additionally, the increased postprandial blood glucose values prior to seroconversion suggest that there is an insult to the pancreas and/or an intrinsic deficit in glucose disposition that contributes to the eventual autoimmunity. Other evidence for an insult includes the associations with prior virus infection (32–34) and type 1 interferon signatures in children who develop islet autoimmunity (35). The manifestation of autoantibodies is likely to occur subsequent to T cell priming to islet β cells. Autoantibodies are likely, but not necessarily, a true indication of the timing of the autoimmune activation. We were unable to discern when T cell priming occurred in children who developed islet autoantibodies in the POInT study, but have previously shown that CD4^+^ T cell responses to proinsulin or GAD65 can be detected around the time of insulin and GAD65 autoantibody seroconversion, respectively (36).

The temporal relationship between blood glucose alterations and islet autoantibody seroconversion further fuels the discussion on the role of autoimmunity versus the pancreas in the pathogenesis and clinical manifestation of T1D (37). There is now increasing evidence for a role of intrinsically or extrinsically derived changes within the pancreas in the initiation of autoimmunity and/or progression to disease (30, 37–40). Nevertheless, there is also ample evidence of increased numbers of autoantigen-targeting CD4^+^ and CD8^+^ T cells within pancreatic islets of patients (41–43). Importantly, immunological interventions have successfully delayed disease manifestation (44) or preserved β cell function (45–47), indicating that autoimmunity is likely to be a significant contributor to late loss of β cells and/or β cell function.

Our findings are also relevant to early programming of type 2 diabetes. The glucose GRS used in our study is associated with type 2 diabetes risk (2, 48). The findings that a higher glucose GRS was associated with higher pre- and postprandial blood glucose in very early childhood and that the greatest effects were at the age of blood glucose nadir are indicative that pancreatic islet function in people who develop type 2 diabetes may already be compromised at 1 year of age. Although childhood BMI and preadolescent weight are also associated with type 2 diabetes risk (49), we observed no relationship between the glucose GRS and BMI in early childhood, suggesting that the GRS affects glucose and type 2 diabetes risk independently of BMI.

The study has some limitations. It is possible that additional or different findings would be observed if fasting blood glucose and standardized food intake were used for pre- and postprandial measurements, but these measurements were not considered feasible in this age group at the time of developing the study protocol. Although the cohort is relatively large, there were only 77 children with positive islet autoantibody outcomes. Moreover, the majority of these outcomes occurred during the more intense period of both pre- and postprandial glucose measurement and 25% occurred after age 1.5 years, when preprandial measurements were performed at 6 monthly intervals and postprandial glucose values were no longer measured. Therefore, the observed relationship between blood glucose and islet autoimmunity may be limited to early onset autoimmunity. The findings were generated in European children with a greater than 25-fold elevated risk of developing T1D, and thus the findings may not be representative of children without an elevated T1D risk or children from other ethnic and racial groups and may not be representative of islet autoimmunity that occurs later in life. An important consideration is that the children were randomized 1:1 to receive placebo or oral insulin at doses that were increased to 67.5 mg daily before 1 year of age with continuation at this dose until 3 years of age. Previous studies showed no effect on blood glucose prior to or after the intake of oral insulin at these doses, consistent with the expectation that the insulin is digested relatively quickly following ingestion (50–53). Moreover, most associations in the current study were already seen for the preprandial blood glucose value measured at the first visit, prior to any administration of the IMP, and the preprandial measurements at subsequent visits were performed 1 day after the last administration of the IMP. Nevertheless, we cannot exclude the possibility that, with larger numbers of children, such as in this study, the IMP may have a small effect on blood glucose in some individuals.

This longitudinal study of blood glucose measurements from infancy has uncovered surprising dynamics and heterogeneity in blood glucose values in early childhood as well as relationships between impaired blood glucose control and the onset of islet autoimmunity. The findings that the metabolic component of T1D pathogenesis may be more substantial and earlier than previously considered portend the intriguing possibility that the pancreatic β cell is the etiological source of the autoimmunity. They also highlight the need for a more intensive study of glucose metabolism in early childhood, for example, using continuous glucose monitoring (54), for the identification of markers of islet health or damage (55), and for investigations of the developing human pancreas (56), so that strategies can be developed to improve metabolism and reduce the incidence of childhood T1D.

## Methods

### Participants.

POInT, a randomized controlled trial, has enrolled 1,050 infants aged 4.0 to 7.0 months with elevated genetic risk of developing T1D (10). Infants were eligible if they had an estimated greater than 10% risk of developing multiple islet autoantibodies by the age of 6 years (57, 58). High genetic risk for infants without a first-degree family history of T1D was defined as a DR3/DR4-DQ8 or DR4-DQ8/DR4-DQ8 genotype and a GRS greater than 14.4. The GRS was calculated as previously described, by multiplying the number of risk alleles (i.e., 0, 1, or 2 for each SNP) with the weight assigned to each SNP tag (Supplemental Table 7). The weighted contributions of all SNPs plus an additive constant of 3.15 for the HLA DR4-DQ8/DR4-DQ8 genotype or 3.98 for the HLA DR3/DR4-DQ8 genotype were summed. For infants with a first-degree family history of T1D, high genetic risk was defined as having HLA DR4 and DQ8 and neither of the protective alleles DRB1*1501 or DQB1*0503.

The children were randomized to receive either oral insulin powder or oral placebo powder daily until 36 months of age. Randomization occurred after the introduction of solid food into the child’s diet. The trial is being conducted at 7 clinical study sites, including 3 in Germany (Munich, Dresden, Hanover), 1 in Poland (Warsaw), 1 in Sweden (Malmö), 1 in Belgium (Leuven), and 1 in the United Kingdom (Oxford). Blood samples are collected during study visits at baseline (4–7 months of age); after 2, 4, and 8 months of the study; at 18 months of age; and then every 6 months until 7.5 years of age. Blood samples for random glucose determinations are collected in the morning (fasting is not required) before IMP intake. At visits 1–4, blood glucose samples are also collected at 30 and 60 minutes after IMP intake and are available for analysis. IMP powder (placebo or insulin) is given together with a small quantity of food, such as breast milk, infant formula or homogenates, bread, yogurt, or other foods. Previous studies suggest that oral insulin administration does not affect blood glucose values or glucose metabolism because insulin is likely to be digested before it can be absorbed (50–53). POInT is organized through the Global Platform for the Prevention of Autoimmune Diabetes (GPPAD) (59).

### Laboratory measurements.

Venous glucose concentrations were measured locally at certified hospital laboratories (enzymatic reference method with hexokinase) or by HemoCue in the UK and Sweden (HemoCue 201 system). *INS* SNP rs689 typing was performed at a central genotyping laboratory (LGC Group; https://www.lgcgroup.com) using DNA extracted from dried blood spots as part of the previously described eligibility criteria (58). The glucose GRS was obtained from SNP data for 16 SNPs previously associated with fasting glucose homeostasis in adult nondiabetic people of European ancestry (48) and calculated according to Dupuis et al. (2). The 16 SNPs and their relative weights are listed in Supplemental Table 2. SNP data were generated using the Infinium Global Screening Array (version 3.0, Illumina Inc.) performed on DNA extracted from dried blood spot samples of children for whom consent to store and use dried blood spots for additional research was provided. Samples were excluded if the genotype call rate was less than 95%, if there was a mismatch between genotyped sex and reported sex, or if there was an outlying heterozygosity rate (>3 SD at a minor-allele frequency [MAF] of <1% or MAF of ≥1%). Variants were filtered if the call rate was less than 98% or if the MAF was greater than 1%. Imputation of additional variants was performed using the Sanger Imputation Service (https://imputation.sanger.ac.uk/) and the Haplotype Reference Consortium reference panel (http://www.haplotype-reference-consortium.org/) HRC r1.1 2016 (GRCh3/hg19).

### BMI.

The children’s heights and weights were obtained at the GPPAD sites by trained personnel at each visit. Height was measured in centimeters as length before 2 years of age and as the standing height measured to the nearest 0.1 cm from 2 years of age using a wall-mounted stadiometer. Body weight was measured in kilograms using regularly calibrated electronic scales.

### Islet autoantibodies.

Islet autoantibodies were measured centrally at 2 independent GPPAD Core laboratories, located at the Institute of Diabetes Research, Helmholtz Munich, Germany, and at the University of Bristol Medical School, Diabetes and Metabolism, Learning and Research, Southmead Hospital, Bristol, United Kingdom (for confirmation of results). Serum samples from each visit were measured for autoantibodies against insulin (IAA), glutamate decarboxylase-65 (GADA), insulinoma-associated antigen-2 (IA-2A), and zinc transporter-8 (ZnT8A) at the German laboratory. IAA were detected using a competitive radiobinding assay (RBA) with protein A/G immunoprecipitation and ^125^I-labeled recombinant human insulin (60). GADA and IA-2A were measured based on the National Institute of Diabetes and Digestive and Kidney Diseases (NIDDK) harmonized assay protocol using ^35^S-methionine–labeled antigens produced by in vitro transcription and translation of N-terminally truncated GAD65 (amino acids 96–585) or IA-2ic (amino acids 606–979), encoded in the pTNT plasmid vector (Promega), as previously described (61). For GADA, ELISA (RSR Ltd.) was used as the second test if the RBA result was positive. ZnT8A was tested in separate assays to detect autoantibodies to the arginine 325R and tryptophan 325W human ZnT8 variants (ZnT8RA and ZnT8WA, respectively). Assays were performed using ^35^S-methionine–labeled in vitro transcribed/translated recombinant ZnT8 (amino acids 268–369), as previously described (62). Children were classified as ZnT8A positive if they were positive for ZnT8RA and/or ZnT8WA or as ZnT8A negative if the tests were negative for both antibody specificities. These RBAs had sensitivities and specificities of 54% and 99% for IAA, 66% and 99% for GADA, 76% and 100% for IA-2A, 56% and 99% for ZnT8RA, and 50% and 99% for ZnT8WA according to the Islet Autoantibody Standardization Program (IASP) 2016 Workshop (63).

Samples that tested positive for islet autoantibodies at the Munich laboratory were sent to the second central autoantibody laboratory in Bristol for confirmatory testing. Here, IAA were assayed using a competition RBA with ^125^I-labeled human insulin, as previously described (64, 65). GADA and IA-2A were assayed using the NIDDK harmonized assay protocol using ^35^S-methionine–labeled in vitro transcribed/translated recombinant full-length GAD65 or IA-2ic (61). ZnT8RA and ZnT8WA were tested in separate RBAs based on the NIDDK harmonized assay protocol (66). These RBAs had sensitivities and specificities of 54% and 99% for IAA, 74% and 97% for GADA, 70% and 100% for IA-2A, 60% and 100% for ZnT8RA, and 46% and 100% for ZnT8WA according to the IASP 2015 Workshop (63).

If a sample tested positive for a specific autoantibody by tests at both laboratories, a subsequent sample was tested by both laboratories to confirm persistence of the islet autoantibody–positive status for the detected autoantibody.

### Definition of islet autoantibody status.

A child was classified as islet autoantibody positive if 2 consecutive samples tested positive at both laboratories. A child was classified as being multiple islet autoantibody positive if they tested positive for 2 or more autoantibodies in 2 consecutive samples in both laboratories. Maternally transferred islet autoantibodies were identified if the child was positive at their first sample and subsequently became islet autoantibody negative and were not included in the classification of islet autoantibody status of the child. For children classified as islet autoantibody positive, the first positive sample was taken as the age at seroconversion.

### Data availability.

Data will be available with a signed transfer agreement; please email cc@gppad.org and the corresponding author.

### Statistics.

Data were extracted from the clinical trial database in October 2021. Glucose measurements were harmonized across study sites by adjusting concentrations by the respective site estimate obtained from a linear regression model. Linear regression models were used to correlate the site-adjusted pre- and postprandial glucose values to the current age of the children. Additionally, generalized additive models (67) were used to display more dynamic effects. Factors that potentially influence glucose values, such as sex, BMI, genetic data (genes associated with blood glucose and *INS*), T1D family history, and islet autoantibody status, were included in the models. Sensitivity analyses were performed by omitting measurements from the United Kingdom and Sweden because these sites used the HemoCue method to measure blood glucose. BMI and glucose GRS were used as continuous variables or expressed as values above or below the median of all values. Student’s *t* test was used to determine whether the regression coefficient was different from zero. For all comparisons, a 2-tailed *P* value of 0.05 after Bonferroni’s correction was considered significant. All data analyses were conducted using R software (2020, https://www.R-project.org/).

### Study approval.

POInT was approved by the institutional review boards and regulatory authorities in each country. In particular, the study was approved by the local ethical committees and regulatory authorities of the Technische Universität München, Medical Faculty (326/17 Af), the Medical University of Warsaw (199/2017), the UK Health Research Authority (18/SC/0019), Onderzoek UZ/KU Leuven (S60711), and Regionala Etikprövningsnämnden i Lund (2017/918). Written, informed consent to participate in the study was obtained from all study participants or their legal representatives. A separate informed consent from the participating families was required to allow biobank storage of material such as DNA that was used in this study.

## Author contributions

KW, AW, EB, and AGZ conceived the analyses, performed data analyses, and drafted the manuscript. KH supported the generation of glucose SNP data. AGZ is the principal investigator of POInT and the speaker of the GPPAD consortium and GPPAD coordination center. PA, TVDB, RB, KC, LG, AH, OK, HEL, ML, BAM, MDS, AS, and JAT are clinical site investigators and contributed to participant enrollment and study conduct. All authors reviewed and approved the manuscript.

## Supplementary Material

Supplemental data

## Figures and Tables

**Table 1:**
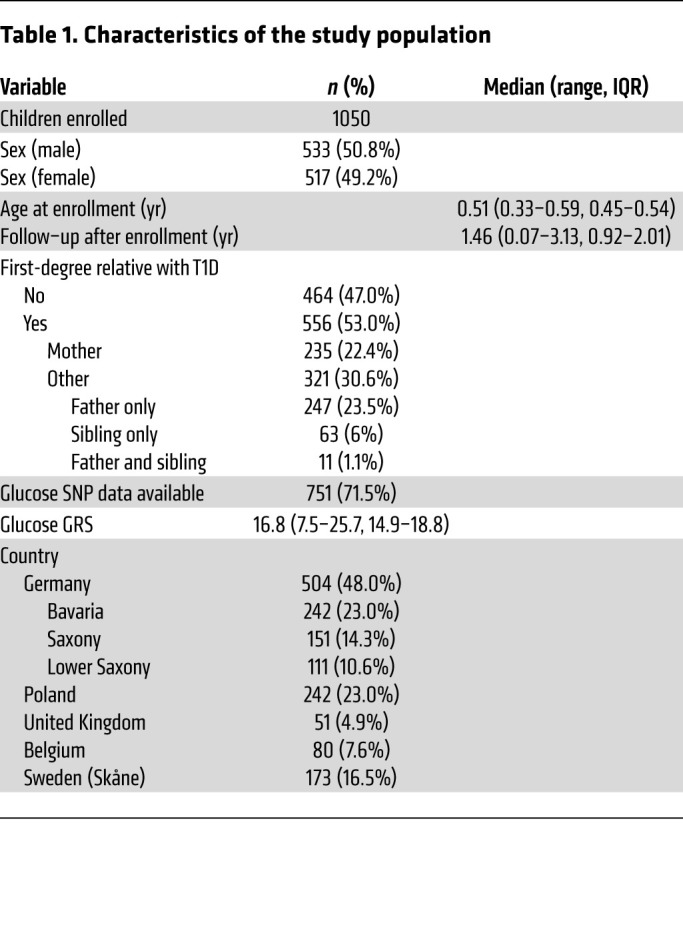
Characteristics of the study population

**Table 2:**
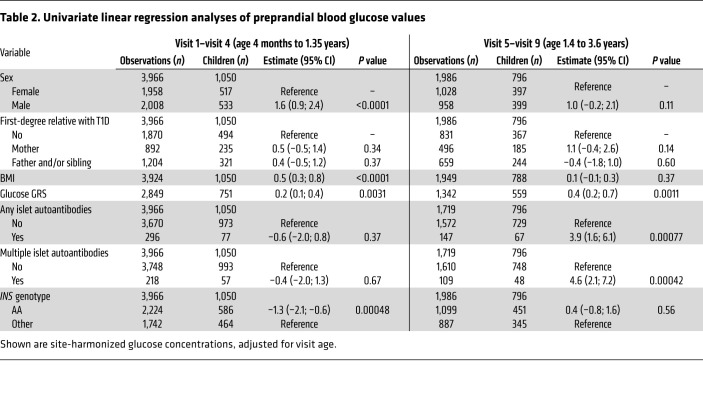
Univariate linear regression analyses of preprandial blood glucose values

**Table 3:**
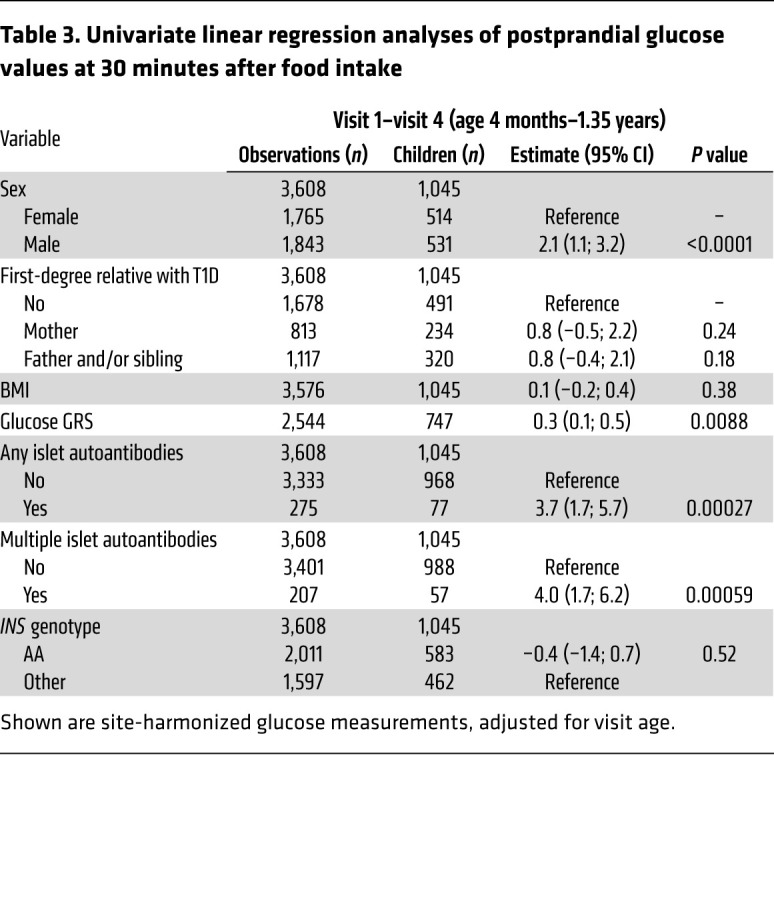
Univariate linear regression analyses of postprandial glucose values at 30 minutes after food intake

## Version 1 10/17/2022

Electronic publication
