# Supplementary material for: Elevations in blood glucose before and after the appearance of islet autoantibodies in children
Source: J Clin Invest. 2022 Oct 17;132(20):e162123. doi: 10.1172/JCI162123 (PMC9566912; doi:10.1172/JCI162123)
Supplement: Supplemental data [file jci-132-162123-s026.pdf]

Elevations in blood glucose before and after the appearance of islet autoantibodies in children

Katharina Warncke[1,2], Andreas Weiss[1], Peter Achenbach[1,3,4], Thekla von dem Berge[5], Reinhard Berner[6], Kristina Casteels[7,8], Lidia Groele[9], Konstantinos Hatzikotoulas[10], Angela Hommel[11,12], Olga Kordonouri[5], Helena Elding Larsson[13,14], Markus Lundgren[13,15], Benjamin A. Marcus[1,3], Matthew D. Snape[16], Agnieszka Szypowska[17], John A. Todd[18], Ezio Bonifacio[11,12], Anette-G. Ziegler[1,3,4] for the GPPAD and POInT Study Groups*

[1] Institute of Diabetes Research, Helmholtz Munich, German Center for Environmental Health, Munich, Germany

[2] Department of Pediatrics, Kinderklinik München Schwabing, School of Medicine, Technical University of Munich, Munich, Germany

[3] Forschergruppe Diabetes, School of Medicine, Klinikum rechts der Isar, Technical University Munich, Munich, Germany

[4] Forschergruppe Diabetes e.V. at Helmholtz Munich, German Research Center for Environmental Health, Munich, Germany

[5] Kinder- und Jugendkrankenhaus AUF DER BULT, Hannover, Germany

[6] Department of Pediatrics, University Hospital Carl Gustav Carus, Technische Universität Dresden, Dresden, Germany

[7] Department of Pediatrics, University Hospitals Leuven, Leuven, Belgium

[8] Department of Development and Regeneration, KU Leuven, Leuven, Belgium

[9] Department of Paediatrics, The Children's Clinical Hospital Józef Polikarp Brudziński, Warsaw, Poland

[10] Institute of Translational Genomics, Helmholtz Munich, German Research Center for Environmental Health, Neuherberg, Germany

[11] Technische Universität Dresden, Center for Regenerative Therapies Dresden, Dresden, Germany

[12] Paul Langerhans Institute Dresden of the Helmholtz Munich at University Hospital Carl Gustav Carus and Faculty of Medicine, TU Dresden, Germany

[13] Unit for Pediatric Endocrinology, Department of Clinical Sciences Malmö, Lund University, Malmö, Sweden

[14] Department of Paediatrics, Skåne University Hospital, Malmö, Sweden

[15] Department of Pediatrics, Kristianstad Hospital, Kristianstad, Sweden

[16] Oxford Vaccine Group, University of Oxford Department of Paediatrics, and NIHR Oxford Biomedical Research Centre, University of Oxford, Oxford, UK

[17] Department of Paediatrics, Medical University of Warsaw, Warsaw, Poland

[18] Wellcome Centre for Human Genetics, Nuffield Department of Medicine, NIHR Oxford Biomedical

Supplemental material

Table of Contents

Supplemental Table 1………………………………………………………………………..4

Supplemental Table 2………………………………………………………………………..5

Supplemental Table 3………………………………………………………………………..6

Supplemental Table 4………………………………………………………………………..7

Supplemental Table 5………………………………………………………………………..8

Supplemental Table 6………………………………………………………………………..9

Supplemental Table 7………………………………………………………………………..10

Supplemental Figure 1………………………………………………………………………12

Supplemental Figure 2………………………………………………………………………13

Supplemental Figure 3………………………………………………………………………14

Supplemental Figure 4………………………………………………………………………15

Supplemental Acknowledgements…………………………………………………………...16

Supplemental Table 1. Visit age and mean non-fasting pre- and post-prandial blood glucose values

Visit	N children with glucose measurement	Age at visit; Median (Min – Max) (years)	Mean pre-prandial glucose concentration (mg/dl (mmol/L))	Mean 30 min. post prandial glucose concentration (mg/dl(mmol/L))	Mean 60 min. post prandial glucose concentration (mg/dl (mmol/L))
Visit 1	1,038	0.51 (0.33 – 0.59)	92.6 (5.14)	105.0 (5.83)	100.8 (5.60)
Visit 2	1,014	0.67 (0.48 – 0.80)	90.6 (5.03)	104.8 (5.82)	99.0 (5.50)
Visit 3	1,003	0.83 (0.64 – 0.99)	89.1 (4.95)	103.0 (5.72)	98.4 (5.47)
Visit 4	911	1.16 (0.94 – 1.34)	88.7 (4.93)	99.6 (5.53)	96.9 (5.38)
Visit 5	775	1.50 (1.39 – 1.69)	88.1 (4.89)	-	-
Visit 6	590	1.99 (1.75 – 2.21)	89.4 (4.97)	-	-
Visit 7	354	2.49 (2.41 – 2.73)	90.4 (5.02)	-	-
Visit 8	194	3.00 (2.94 – 3.17)	91.4 (5.08)	-	-
Visit 9	73	3.46 (3.39 – 3.60)	92.6 (5.14)	-	-

Supplemental Table 2. SNPs and their respective weights used to calculate the glucose GRS

SNP	Risk allele	Nearest gene	Allele Weight
Rs340874	C	PROXI	0.439
Rs11071657	A	FAM148B	0.270
Rs11558471	A	SLC30A8	0.911
Rs4506565	T	TCF7L2	0.885
Rs560887	C	G6PC2	2.532
Rs10830963	G	MTNR1B	2.262
Rs4607517	A	GCK	2.093
Rs2191349	T	DGKB/TMEM195	1.013
Rs780094	C	GCKR	0.979
Rs11708067	A	ADCY5	0.911
Rs7944584	A	MADD	0.709
Rs10885122	G	ADRA2A	0.743
Rs174550	T	FADS1	0.574
Rs11605924	A	CRY2	0.506
Rs11920090	T	SLC2A2	0.675
Rs7034200	A	GLIS3	0.608

SNP, single nucleotide polymorphism; GRS, genetic risk score

Table 3. Multivariate linear regression analysis (site-harmonized glucose measurements, adjusted for visit age)

	Visit 1 – Visit 4 (age 4 months – 1.35 years)				Visit 5 – Visit 7 (age 1.4 years – 2.7 years)			
Variable	N observations	N children	Estimate [95% CI]	P-value	N observations	N children	Estimate [95% CI]	P-value
**Gender**	2,835	751			1,182	559	Not included	-
Female	1,419	373	Reference	-	619	285		
Male	1,416	378	1.2 [0.3; 2.1]	0.0099	563	274		
**BMI**	2,835	751	0.4 [0.1; 0.7]	0.0029	1,182	559	Not included	-
**Glucose GRS**	2,835	751	0.2 [0.1; 0.4]	0.0016	1,182	559	0.4 [0.2; 0.7]	0.0011
**Any islet autoantibodies**	2,835	751	Not included	-	1,182	559		
No	2,662	706			1,105	521	Reference	
Yes	173	45			77	38	4.30 [1.2; 7.2]	0.006
**INS genotype**	2,835	751			1,182	559	Not included	-
AA	1,603	425	-1.4 [-2.3; -0.5]	0.0017	652	317		
other	1,232	326	Reference		530	242		

BMI Body Mass Index, GRS Genetic Risk Score, INS Insulin

Supplemental Table 4**.** Multivariate linear regression analyses (site-harmonized glucose measurements, adjusted for visit age) of post prandial glucose values at 30 min after food intake

	Visit 1 – Visit 4 (age 4 months – 1.35 years)			
Variable	N observations	N children	Estimate (95% CI)	P-value
**Sex**	2,544	747		
Female	1,249	370	Reference	–
Male	1,295	377	2.4 (1.1; 3.6)	0.0002
**Glucose GRS**	2,544	747	0.3 (0.1; 0.5)	0.003
**Any islet autoantibodies**	2,544	747		
No	2,429	715	Reference	
Yes	115	32	5.4 (2.4; 8.4)	0.0004

CI, confidence interval; GRS, genetic risk score;

Supplemental Table 5. Univariate linear regression analyses (site-harmonized pre-prandial glucose measurements, adjusted for visit age): sensitivity analysis excluding data from UK and Sweden.

	Visit 1 – Visit 4				Visit 5 – Visit 9*			
Variable	N observations	N children	Estimate (95% CI)	P-value	N observations	N children	Estimate (95% CI)	P-value
**Sex**	3,124	826			1,609	620		
Female	1,566	412	Reference	–	847	314	Reference	–
Male	1,558	414	1.4 (0.7; 2.1)	<0.0001	762	306	1.1 (−0.2; 2.4)	0.085
**First degree relative with T1D**	3,124	826			1,609	620		
No	1,348	355	Reference	–	612	258	Reference	–
Mother	753	199	0.7 (−0.2; 1.6)	0.14	427	156	0.9 (−0.7; 2.5)	0.27
Other	1,023	272	1.0 (0.2; 1.9)	0.012	570	206	−0.8 (−2.3; 0.7)	0.28
**BMI**	3,121	826	0.6 (0.4; 0.8)	<0.0001	1,605	620	0.8 (0.3; 1.2)	0.00051
**Glucose GRS**	2,233	588	0.2 (0.1; 0.3)	0.0040	1,069	433	0.4 (0.1; 0.6)	0.0066
**Any islet autoantibodies**	3,124	826			1,360	620		
No	2,882	763	Reference		1,235	566	Reference	
Yes	242	63	−0.1 (−1.4; 1.3)	0.91	125	54	2.2 (−0.1; 4.5)	0.059
**Multiple islet autoantibodies**	3,124	826			1,360	620		
No	2,952	781	Reference		1,269	583	Reference	
Yes	172	45	0.1 (−1.5; 1.6)	0.93	91	37	3.0 (0.3; 5.7)	0.028
***INS* genotype**	3,124	826			1,609	796		
AA	1,756	463	−1.1 (−1.8; −0.4)	0.0024	892	352	0.2 (−1.1; 1.5)	0.77
other	1,368	363	Reference		717	268	Reference	

CI, confidence interval; T1D, type 1 diabetes; BMI, body mass index; GRS, genetic risk score; *INS*, *Insulin* gene

Supplemental Table 6. Univariate linear regression analyses (site-harmonized glucose measurements, adjusted for visit age): sensitivity analysis using pre-prandial glucose values at visit 1 only

	Visit 1 (Age 4 – 7 months)		
Variable	N observations	Estimate (95% CI)	P-value
**Sex**	1,038		
Female	511	Reference	–
Male	527	1.8 (0.4; 3.2)	0.013
**First degree relative with T1D**	1,038		
No	489	Reference	–
Mother	231	−0.73 (−2.6; 1.1)	0.44
Other	318	0.05 (−1.6; 1.7)	0.94
**BMI**	1,038	1.1 (0.7; 1.5)	<0.0001
**Glucose GRS**	743	0.2 (−0.1; 0.5)	0.17
**Any islet autoantibodies**	1,038		
No	966	Reference	
Yes	72	−0.2 (−3.1; 2.6)	0.88
**Multiple islet autoantibodies**	1,038		
No	985	Reference	
Yes	53	0.5 (−2.7; 3.8)	0.76
***INS* genotype**	1,038		
AA	580	−2.3 (−3.8; −0.9)	0.0016
other	458	Reference	

CI, confidence interval; T1D, type 1 diabetes; BMI, body mass index; GRS, genetic risk score; *INS, Insulin* gene

Supplemental Table 7: List of SNPs used to determine study eligibility and risk score calculation

SNP	Gene, Allele, or Haplotype	Score weight for genotype or per allele
**HLA class II**		
rs17426593	HLA DR4-DQ8/DR4-DQ8	3.15
rs2187668		3.98
rs7454108	HLA DR3/DR4-DQ8	
rs3129889	HLA *DRB1**1501	Exclusion criteria for first degree relatives
rs1794265	HLA *DQB1**0503	Exclusion criteria for first degree relatives
**HLA class I**		
rs1264813	HLA A 24	0.43
rs2395029	HLA B 5701	0.92
**Non-HLA SNPs**		
rs2476601	*PTPN22*	0.76
rs2816316	*RGS1*	0.16
rs3024505	*IL10*	0.22
rs1990760	*IFIH1*	0.16
rs3087243	*CTLA4*	0.16
rs10517086	*C4orf52*	0.19
rs2069763	*IL2*	0.11
rs6897932	*IL7R*	0.19
rs3757247	*BACH2*	0.19
rs9388489	*C6orf173*	0.14
rs6920220	*TNFAIP3*	0.15
rs1738074	*TAGAP*	0.05
rs7804356	*SCAP2*	0.15
rs4948088	*COBL*	0.17
rs7020673	*GLIS3*	0.23
rs12722495	*IL2RA*	0.47
rs947474	*PRKCQ*	0.15
rs10509540	*RNLS/C10orf59*	0.25
rs689	*INS*	0.65
rs4763879	*CD69*	0.06
rs2292239	*ERBB3*	0.36
rs3184504	*SH2B3*	0.24
rs1465788	*ZFP36L1*	0.13
rs17574546	*RASGRP1*	0.13

rs3825932	*CTSH*	0.15
rs12708716	*CLEC16A*	0.15
rs4788084	*IL27*	0.20
rs7202877	*CTRB2*	0.19
rs2290400	*ORMDL3*	0.25
rs7221109	*CCR7*	0.15
rs45450798	*PTPN2*	0.09
rs763361	*CD226*	0.12
rs425105	*PRKD2*	0.21
rs2281808	*SIRPG*	0.07
rs3788013	*UBASH3a*	0.16
rs5753037	*RPS3AP51*	0.15
rs229541	*IL2B*	0.18
rs5979785	*TLR8*	0.09
rs2664170	*GAB3*	0.14

The risk score is calculated by multiplying the number of risk alleles (i. e. 0, 1 or 2 for each single SNP) with the weight assigned to each SNP and then summing up the weighted contributions of all SNPs plus an additive constant of 3.15 for infants who have the HLA DR4-DQ8/DR4-DQ8 genotype or 3.98 for infants who have the HLA DR3/DR4-DQ8 genotype. As an example, the risk score for a child with HLA DR4-DQ8/DR4-DQ8, homozygous for the risk allele of rs1264813 (weight 0.43), heterozygous for the risk allele of rs2395029 (weight 0.92), homozygous for the non-risk allele of rs2476601 (weight 0.76) and for all other SNPs in the genetic risk score is calculated as follows:

Risk score = 3.15 + (2 * 0.43) + (1 * 0.92) + (0 * 0.76) + 0 = 4.93

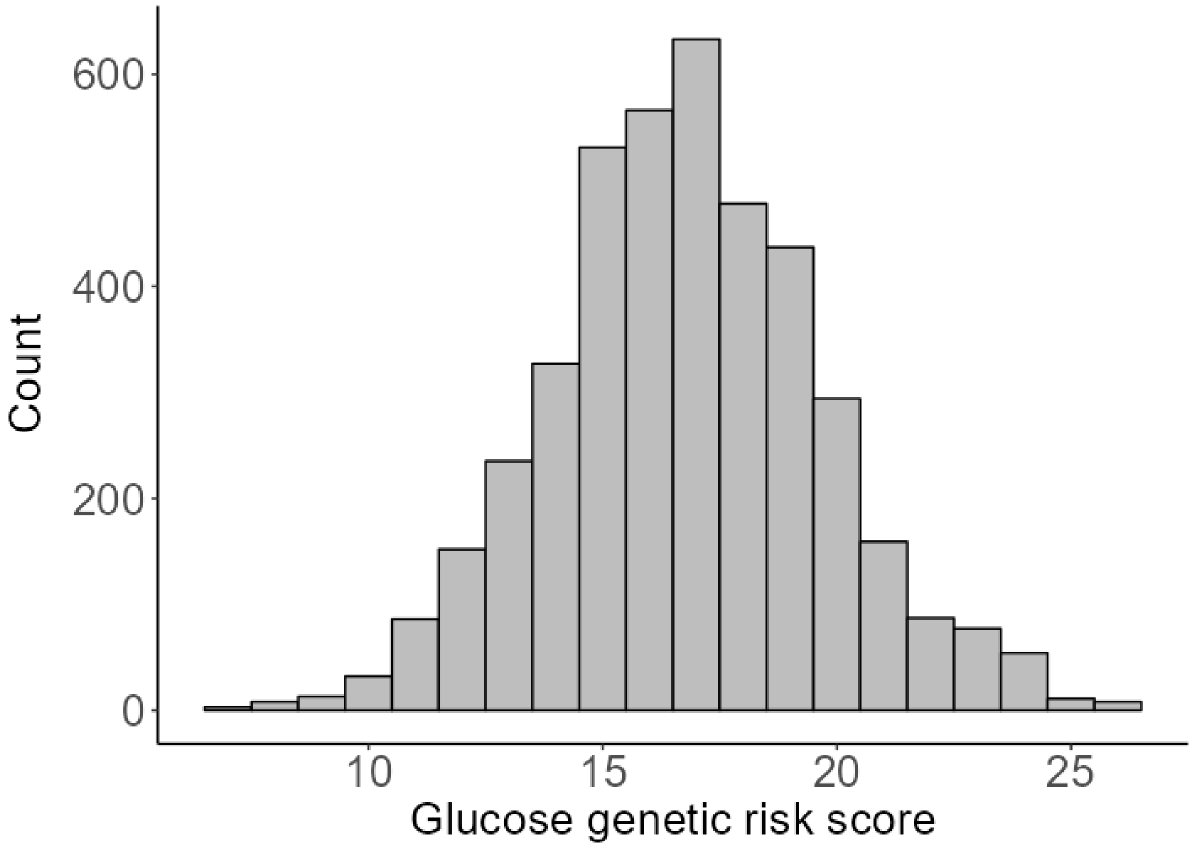

Supplemental Figure 1. Histogram showing the distribution of the glucose genetic risk score (count of measurements for each unit of score).

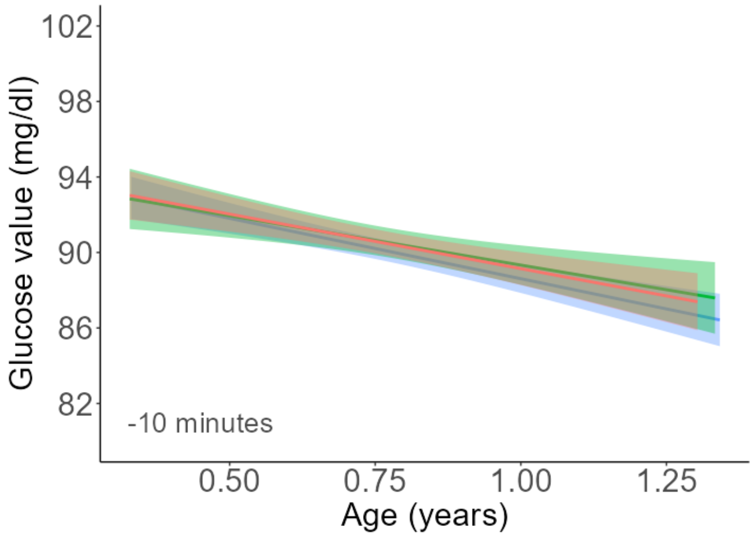

Supplemental Figure 2. Linear regression of pre-prandial glucose values by visit age in the infancy period (visits 1–4; 4 months–1.35 years of age) according to the child's first-degree relative status: none (blue), mother (green), and other (red).

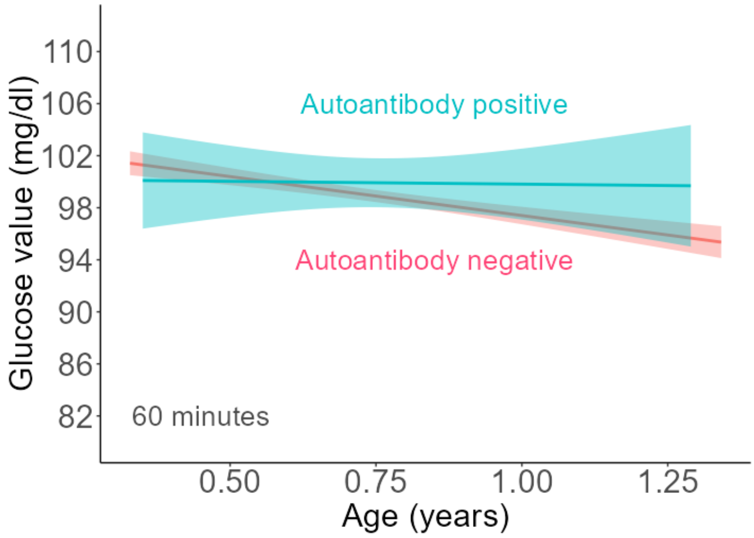

Supplemental Figure 3. Linear regression of post-prandial glucose values (60 min after food intake) by visit age in the infancy period (visits 1– 4; 4 months–1.35 years of age) for islet autoantibody-positive (blue) and islet autoantibody-negative (red) children.

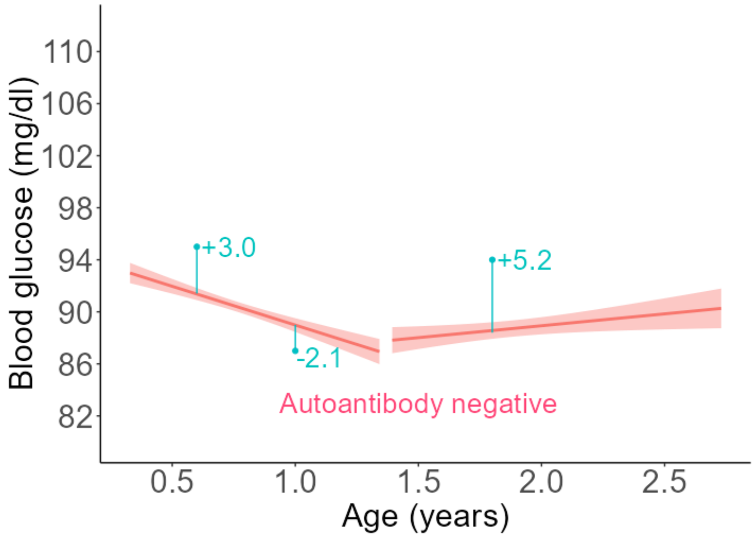

Supplemental Figure 4. Schematic of the method used to calculate the differences in glucose values in autoantibody positive children (blue points) from the linear regression curves of glucose values in autoantibody negative-children (red)

Supplemental Acknowledgements

GPPAD Coordinating Center: Melanie Gündert, Florian Haupt, Stefanie Arnolds, Miriam Bißbort, Karina Blasius, Nadine Friedl, Cigdem Sanverdi, Gertrud Göppel, Martin Heigermoser, Bianca Höfelschweiger, Manja Jolink, Krisztian Kisfügedi, Nadine Klein, Ramona Lickert, Claudia Matzke, Kim Muñoz Alvarez, Rebecca Niewöhner, Marlon Scholz, Katharina Schütte-Borkovec, Franziska Voß, Andreas Weiß, José Maria Zapardiel Gonzalo, Sarah Schmidt, Philipp Sifft, Heidi Kapfelsberger, Merve Vurucu, Katharina Sarcletti, Melanie Sporreiter, Stefanie Jacobsen, and Ivo Zeller.

Belgium Clinical Center: Kristina Casteels, Charlien Jannsen, Annre Rochtus, An Jacobs, Hilde Morobé, Jasmin Paulus, Brontë Vrancken, Natalie Van den Driessche, Renka Van Heyste, Janne Houben, Leyla Smets, and Veerle Vanhuyse.

Germany, Dresden Clinical Center: Ezio Bonifacio, Reinhard Berner, Sari Arabi, Ruth Blechschmidt, Sevina Dietz, Franziska Ehrlich, Gita Gemulla, Zahra Gholizadeh, Sophie Heinke, Raphael Hoffmann, Angela Hommel, Franziska Andrea Lange, Anja Loff, Robert Morgenstern, Anett Piller, Marc Weigelt, Marie Zielmann, and Nicole Zubizarreta.

Germany, Hanover Clinical Center: Olga Kordonouri, Thomas Danne, Laura Galuschka, Ute Holtkamp, Nils Janzen, Carolin Kruse, Sarah Landsberg, Karin Lange, Erika Marquardt, Felix Reschke, Frank Roloff, Kerstin Semler, Thekla von dem Berge, and Jantje Weiskorn.

Germany, Munich Clinical Center: Anette G. Ziegler, Peter Achenbach, Melanie Bunk, Simone Färber-Meisterjahn, Willi Grätz, Ines Greif, Melanie Herbst, Anna Hofelich, Melina Kaiser, Heidi Kaltenecker, Esra Karapinar, Annika Kölln, Benjamin Marcus, Annette Munzinger, Jasmin Ohli, Claudia Ramminger, Franziska Reinmüller, Veronika Vollmuth, Tiziana Welzhofer, and Christiane Winkler.

Poland Clinical Center: Agnieszka Szypowska, Mariusz Ołtarzewski, Sylwia Dybkowska, Katarzyna Dżygało, Lidia Groele, Katarzyna Kajak, Dorota Owczarek, Katarzyna Piechowiak, Katarzyna Popko, Agnieszka Skrobot, Rafał Szpakowski, Anna Taczanowska, Beata Zduńczyk, and Anna Zych.

Sweden Clinical Center: Helena Elding Larsson, Markus Lundgren, Åke Lernmark, Daniel Agardh, Hanna Samuelsson, Sofie Alstrom Mortin, Carin Andrén Aronsson, Rasmus Bennet, Charlotte Brundin, Susanne Dahlberg, Lina Fransson, Ida Jönsson, Sara Maroufkhani, Zeliha Mestan, Caroline Nilsson, Anita Ramelius, Evelyn Tekum Amboh, Carina Törn, and Ulrika Ulvenhag.

UK, Oxford Clinical Center: Matthew Snape, John A Todd, Genevieve Haddock, Owen Bendor-Samuel, James Bland, Edward Choi, Rachel Craik, Kimberly Davis, Sophia Hawkins, Arancha de la Horra, Yama Farooq, Clare Scudder, Ian Smith, Fenella Roseman, Hannah Robinson, Nazia Taj, Manu Vatish, Louise Willis, Conor Whelan, and Tabitha Wishlade.

Betacell autoantibody laboratories: Institute of Diabetes Research, Helmholtz Zentrum München, Munich, Germany; Bristol Medical School, The University of Bristol, Bristol, UK.

Genotyping Laboratory: Grace London, LGC Ltd., Hertfordshire, UK.

Pharmacovigilance: PHARMALOG, GmbH, Ismaning, Germany.

Data Safety and Monitoring Board: Polly Bingley (University of Bristol, Bristol, UK), Ulrich Heininger (Division of Paediatric Infectious Diseases and Vaccinology, University Children's Hospital, Basel, Switzerland), Markus Pfirrmann (Institut für Medizinische Informationsverarbeitung, Biometrie und Epidemiologie (IBE), Ludwig-Maximilians-Universität, Munich, Germany), Wolfgang Rascher (Department of Pediatrics and Adolescent Medicine, Erlangen, Germany), and Paul Turner (Nuffield Department of Medicine, Medical Science Division, Oxford, UK)
